# Efficacy of a Mer and Flt3 tyrosine kinase small molecule inhibitor, UNC1666, in acute myeloid leukemia

**DOI:** 10.18632/oncotarget.3156

**Published:** 2015-02-10

**Authors:** Alisa B. Lee-Sherick, Weihe Zhang, Kelly K. Menachof, Amanda A. Hill, Sean Rinella, Gregory Kirkpatrick, Lauren S. Page, Michael A. Stashko, Craig T. Jordan, Qi Wei, Jing Liu, Dehui Zhang, Deborah DeRyckere, Xiaodong Wang, Stephen Frye, H. Shelton Earp, Douglas K. Graham

**Affiliations:** ^1^ University of Colorado, Department of Pediatrics, Aurora, CO, USA; ^2^ University of North Carolina, Eshelman School of Pharmacy, Chapel Hill, NC, USA; ^3^ University of Colorado, Department of Medicine, Aurora, CO, USA; ^4^ Children's Hospital Colorado, Department of Pathology, Aurora, CO, USA; ^5^ University of North Carolina, Department of Medicine, Chapel Hill, NC, USA

**Keywords:** Tyrosine kinase inhibitor, acute myeloid leukemia, TAM receptors

## Abstract

Mer and Flt3 receptor tyrosine kinases have been implicated as therapeutic targets in acute myeloid leukemia (AML). In this manuscript we describe UNC1666, a novel ATP-competitive small molecule tyrosine kinase inhibitor, which potently diminishes Mer and Flt3 phosphorylation in AML. Treatment with UNC1666 mediated biochemical and functional effects in AML cell lines expressing Mer or Flt3 internal tandem duplication (ITD), including decreased phosphorylation of Mer, Flt3 and downstream effectors Stat, Akt and Erk, induction of apoptosis in up to 98% of cells, and reduction of colony formation by greater than 90%, compared to treatment with vehicle. These effects were dose-dependent, with inhibition of downstream signaling and functional effects correlating with the degree of Mer or Flt3 kinase inhibition. Treatment of primary AML patient samples expressing Mer and/or Flt3-ITD with UNC1666 also inhibited Mer and Flt3 intracellular signaling, induced apoptosis, and inhibited colony formation. In summary, UNC1666 is a novel potent small molecule tyrosine kinase inhibitor that decreases oncogenic signaling and myeloblast survival, thereby validating dual Mer/Flt3 inhibition as an attractive treatment strategy for AML.

## INTRODUCTION

Though the treatment of acute myeloid leukemia (AML) has significantly improved over the past 30 years, average five-year survival rates are still less than 60% for pediatric patients and are progressively worse for older adult and elderly patients [1, 2]. The high-intensity chemotherapy needed to induce remission in AML is often prohibitive in elderly patients due to excessive mortality [3, 4], and is known to cause concerning long-term toxic side effects, including growth abnormalities, cardiac dysfunction, neurocognitive deficits, and gonadal failure in pediatric patients [5–7]. Novel therapies that effectively achieve cancer cell killing with minimal toxicity to normal cells are urgently needed to improve both patient outcomes and long-term quality of life.

The Mer receptor tyrosine kinase is abnormally expressed on 80–100% of AML patient samples, whereas little or no Mer is expressed in normal bone marrow precursor cells [8, 9]. Mer activation in AML cell lines leads to pro-survival signaling, including phosphorylation of Erk1/2, Akt and Stat6. Inhibition of Mer in AML via RNA interference led to a significant increase in myeloblast apoptosis, decreased colony formation in methylcellulose, and prolonged leukemia-free survival in a murine xenograft model [8]. These findings led us to determine that aberrant Mer expression (rather than activating mutation in the kinase domain) provides a survival advantage in leukemia. Taken together, these data suggest that Mer kinase inhibition may selectively target leukemia cells, while sparing normal bone marrow progenitors.

Internal tandem duplication (ITD) mutations of the FMS-like tyrosine kinase 3 (Flt3) receptor, found in ~20–30% of adult AML patient samples and ~15% of pediatric AML samples, is associated with poor prognosis in both patient populations [10–15]. Specifically, a high ratio of the *FLT3-ITD* mutant allele relative to the wild type allele (> 0.4) has been associated with a markedly poor progression-free survival [16]. Constitutive activation of Flt3 through the ITD mutation leads to downstream activation of pro-survival signaling pathways including Stat5, Akt and Erk1/2 [17, 18]. Given that Flt3 tyrosine kinase inhibitors (TKIs) have been effective in preclinical models of AML, Flt3 inhibition is currently being tested in patients with Flt3-ITD mutations. Unfortunately, resistance to Flt3 targeted TKIs has been reported, including selection for novel point mutations [19, 20]. Interestingly, different Flt3 inhibitors do not appear to have overlapping resistance mutation profiles [21], such that mutations resulting in resistance to one inhibitor may not confer resistance to another. Furthermore, adverse side effects have been reported in patients treated with existing Flt-3 inhibitors, including the recent restrictions on clinical use of ponatinib due to higher than expected rates of arterial thrombosis [22]. Issues with resistance and adverse toxicities demonstrate the need to develop new, more effective therapeutic agents.

Given the high prevalence of expression of both Mer and Flt3 and the data indicating their oncogenic roles in AML, targeting them together is an attractive therapeutic strategy, and could potentially result in better outcomes in high-risk patients, reduced need for chemotherapy in low-risk patients, or a therapeutic option for those who cannot tolerate high-intensity chemotherapy. We have developed UNC1666, a novel Mer and Flt3 targeted small molecule tyrosine kinase inhibitor with therapeutic potential in AML. In this publication, we show that this ATP-binding site competitive small molecule potently and selectively inhibits Mer and Flt3 kinase activation and downstream signal transduction resulting in growth inhibition and apoptosis of AML cell lines and primary patient myeloblasts.

## RESULTS

### UNC1666, a novel dual specific Mer and Flt3 tyrosine kinase inhibitor

**Figure 1:**
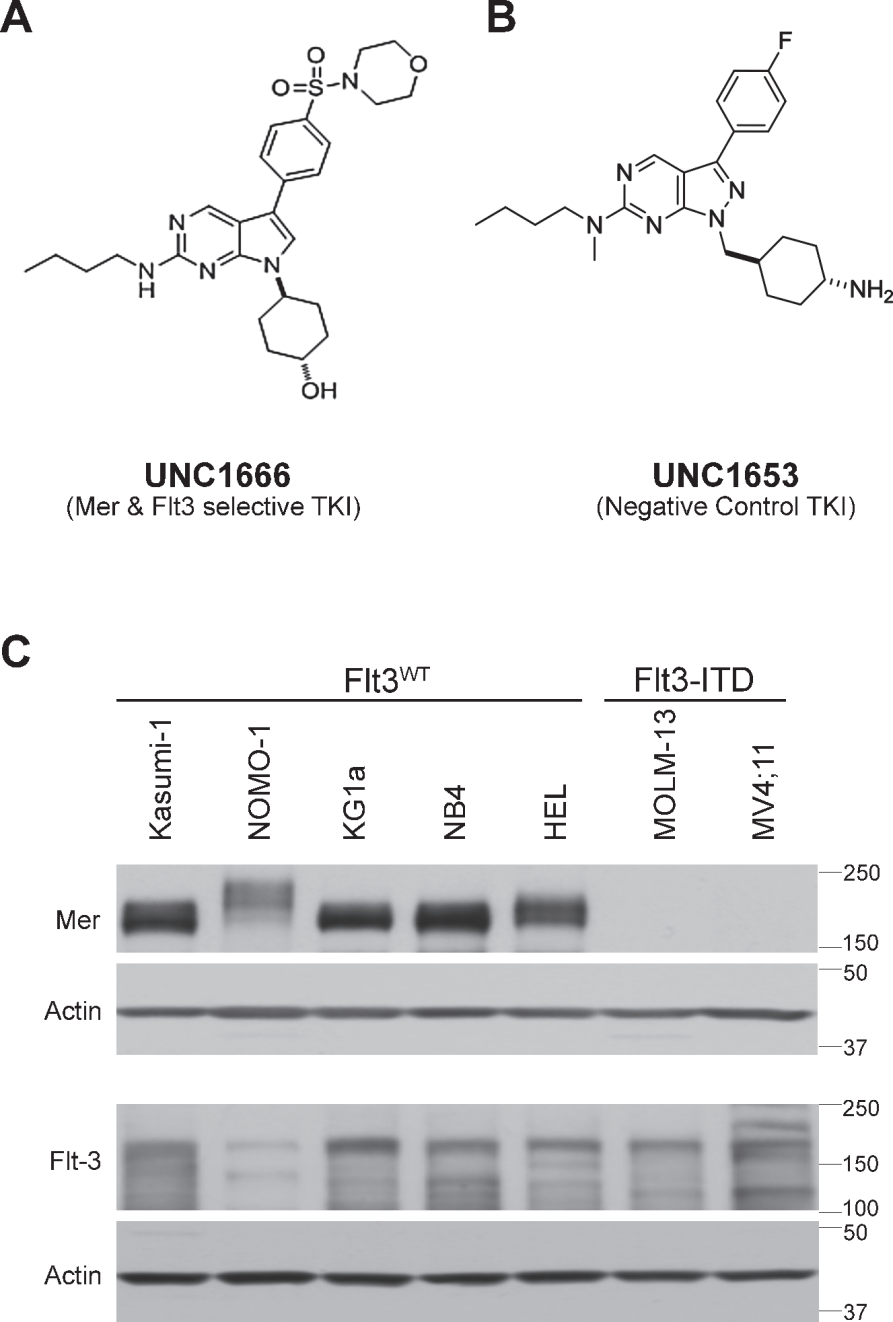
UNC1666 is a novel inhibitor of Mer and Flt3 tyrosine kinases **(A)** Chemical structure of UNC1666, with inhibition constant (K_i_) of 0.16 nM for Mer (enzymatic IC_50_: 0.55 nM) and 0.67 nM for Flt3 (enzymatic IC_50_: 0.69 nM). **(B)** Chemical structure of UNC1653, which lacks significant activity against Mer (enzymatic IC_50_: 560 nM) and Flt3 (enzymatic IC_50_: 220 nM) and is used as a negative control in these studies. **(C)** Whole cell lysates from AML cell lines with known Flt3 mutation status were analyzed by immunoblot and demonstrate presence or absence of the Mer tyrosine kinase (above) and the Flt3 tyrosine kinase (middle). Actin is shown as an indicator of total protein (below).

We previously reported UNC1062 [23], a selective ATP-competitive type I inhibitor of Mer. However, its low solubility and poor *in vivo* pharmacokinetic properties made UNC1062 unsuitable for *in vivo* studies. To develop further Mer inhibitors, a new pyrrolopyrimidine scaffold with better solubility was introduced using a structure-based design approach [24]. UNC1666, a pyrrolopyrimidine analogue with a structure similar to UNC1062, is also an ATP-competitive type I inhibitor (Figure 1A). Analysis of the inhibition constant (K_i_) proved this compound to be more potent and selective for Mer (MCE IC_50_ 0.55 nM; K_i_ 0.16 nM) compared to previously described Mer inhibitors [23, 25]. Additionally, UNC1666 inhibits Flt3 (MCE IC_50_ 0.69 nM; K_i_ 0.67 nM) equipotently in enzymatic MCE assays. A comprehensive protein kinase profiling panel provided by Carna Biosciences was used to assess off-target kinase inhibition mediated by UNC1666 at a concentration of 46 nM, more than 50-fold higher than its MCE IC_50_ values against Mer and Flt3 (Supplemental Table 2). Only the Trk proteins were inhibited greater than 95% in response to treatment with UNC1666. Additional MCE assays were performed to determine inhibition of TrkA (as a surrogate for the Trk family kinases) and revealed similar potency (MCE IC_50_ 0.57 nM) (Supplemental Table 2). Furthermore, we analyzed the effect of UNC1666 on both Tyro-3 and Axl (members of the TAM receptor tyrosine kinase family along with Mer), which demonstrated enzymatic MCE IC_50_ values of 29 nM and 37 nM, respectively.

The small molecule UNC1653 (Figure 1B) has a pyrazolopyrimidine scaffold similar to UNC1062. One of the hinge binding hydrogen bonds was disrupted by introducing a methyl group, which dramatically reduced Mer and Flt3 activity. In these studies, we used UNC1653 as a negative control TKI given that it has weak inhibition of both Mer (MCE IC_50_ 560 nM) and Flt3 (MCE IC_50_ 220 nM) relative to UNC1666.

### UNC1666 reduces Mer and Flt3-mediated anti-apoptotic and pro-survival signaling

We have previously described survival and proliferation signaling resulting from Mer tyrosine kinase activity in AML, including increased activation of Erk1/2, Akt and Stat6 [8]. Similarly, others have demonstrated that Erk1/2, Akt and Stat5 are critical downstream survival and proliferation signals in AML cells with Flt3-ITD mutations [17, 18]. Given the degree of overlap in Mer and Flt3-ITD signaling pathways, we first determined whether UNC1666 could inhibit each kinase independently in AML cell lines. The Kasumi-1 and NOMO-1 cell lines express Mer but do not have activating Flt3 mutations. We previously showed that short hairpin RNA-mediated inhibition of Mer expression in the Kasumi-1 and NOMO-1 cell lines increased stress-induced apoptosis and decreased colony formation [8]. Conversely, we analyzed MV4;11 (homozygous for the Flt3-ITD, high allelic ratio or “Flt3-ITD^highAR^”) and MOLM-13 (heterogygous for the Flt3-ITD, low allelic ratio or “Flt3-ITD^lowAR^”), which express little (“dim”) and no Mer respectively, and have been used extensively as models of leukemia with Flt3-ITD mutations.

**Figure 2:**
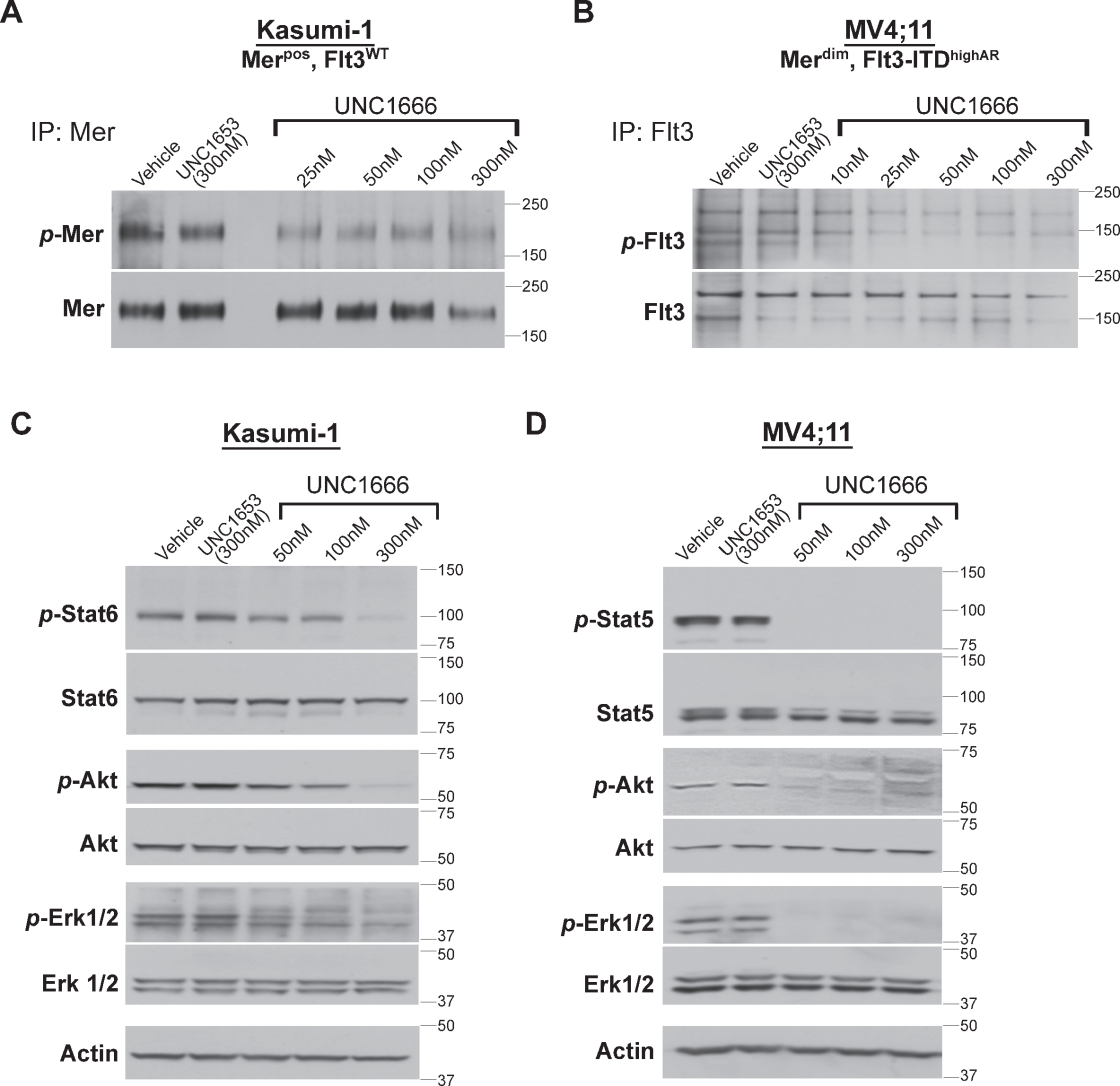
UNC1666 reduces Mer and Flt3-mediated signaling through downstream anti-apoptotic and pro-survival pathways **(A)** Mer was immunoprecipitated from AML cell lysates and phosphorylated (*p*-Mer) and total Mer (~180 kDa) levels were assessed by immunoblot analysis. This representative blot of the Kasumi-1 cell line demonstrates decreased Mer phosphorylation after treatment with increasing doses of UNC1666. **(B)** Flt3 was immunoprecipitated from AML cell lysates and phosphorylated (*p*-Flt3) and total Flt3 (130/160 kDa) levels were assessed by immunoblot analysis. This representative blot of the MV4;11 cell line demonstrates decreased Flt3 phosphorylation after treatment with increasing doses of UNC1666. **(C)** Inhibition of downstream signaling after administration of UNC1666 in a Mer expressing AML cell lines that does not express a Flt3-ITD mutation (Kasumi-1) compared with equivalent concentrations of vehicle (DMSO) or inactive control TKI UNC1653. Actin is shown as an indicator of total protein. **(D)** Downstream signaling after administration of UNC1666 in a Flt3-ITD AML cell line that does not express Mer (MV4;11). Representative blots from at least 3 independent experiments are shown. nM = nanomolar

Using these cell lines, we confirmed that short-term treatment with UNC1666 reduced Mer and Flt3 phosphorylation in a dose dependent manner, compared to vehicle alone (DMSO) or negative control UNC1653 (Figure 2A & 2B). UNC1666 decreased Flt3 phosphorylation more potently in Flt3-ITD cell lines, as compared to Flt3 wild-type cell lines (Figure 2B and data not shown). Mer and Flt3 phospho-protein levels were reduced by approximately half at concentrations of 100 nM and 50 nM, respectively.

To investigate whether inhibition of other kinases might contribute to effects mediated by UNC1666, we also assessed expression of the Trk kinases as well as Tyro-3 and Axl. The cells lines used for these studies express very little or no Tyro-3, Axl or Trk protein (Supplemental Figure 1).

We then evaluated downstream Erk, Akt and Stat signaling after a two hour treatment with UNC1666. There was a consistent dose-dependent abrogation of Erk1/2, Akt and Stat6 phosphorylation in Mer expressing cell lines treated with UNC1666 compared to vehicle or UNC1653 (Figure 2C and Supplemental Figure 2). The decreased phosphorylation of these molecules correlated with the decrease in Mer phosphorylation (Figures 2A and 2C); diminished phosphorylation was noted at 50 nM, a moderate decrease at 100 nM, and more complete inhibition at 300 nM. Additionally, phosphorylation of Erk1/2, Akt and Stat5 was markedly diminished in the Flt3-ITD cell lines after treatment with UNC1666 compared to vehicle or UNC1653 (Figure 2D and Supplemental Figure 2). Again, decreased phosphorylation of these pro-survival signaling molecules in Flt3-ITD cell lines correlated with a decrease in Flt3 phosphorylation (Figures 2B and 2D); near complete ablation of phosphorylation was observed at 50 nM.

### UNC1666 induces apoptosis in AML cell lines

**Figure 3:**
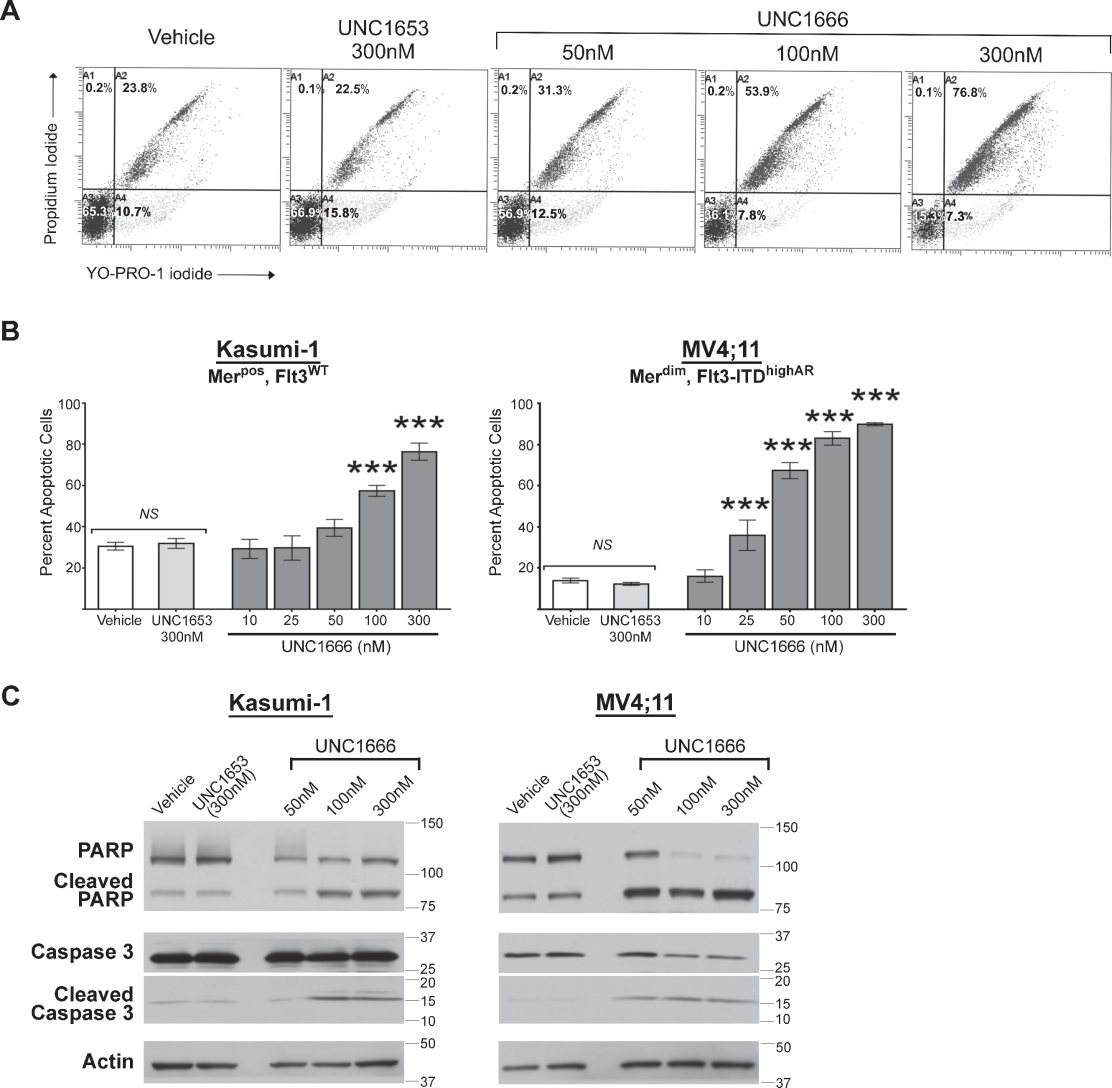
UNC1666 induces apoptosis in myeloblasts expressing Mer or Flt3-ITD Mer^pos^ or Flt3-ITD AML cell lines were treated with UNC1666, vehicle (DMSO), or inactive control TKI UNC1653 for 72 hours and then analyzed by flow cytometry after staining with YO-PRO-1 iodide and propidium iodide to identify apoptotic and dead cells. **(A)** Representative flow cytometry profiles of Kasumi-1 cells are shown. The percentages of live (lower left quadrant), early apoptotic (lower right quadrant), and late apoptotic/dead cells (upper quadrants) are shown. **(B)** Graphic representation of flow cytometric analyses of apoptotic/dead cells. Mean values and standard errors were derived from at least 3 independent experiments. *p < 0.05, ***p < 0.001, *NS* = not significant. **(C)** Cells were treated as indicated for 72 hours, whole cell lysates were prepared and the indicated apoptotic proteins were assessed by immunoblot analysis. Actin is shown as a loading control.

**Figure 4:**
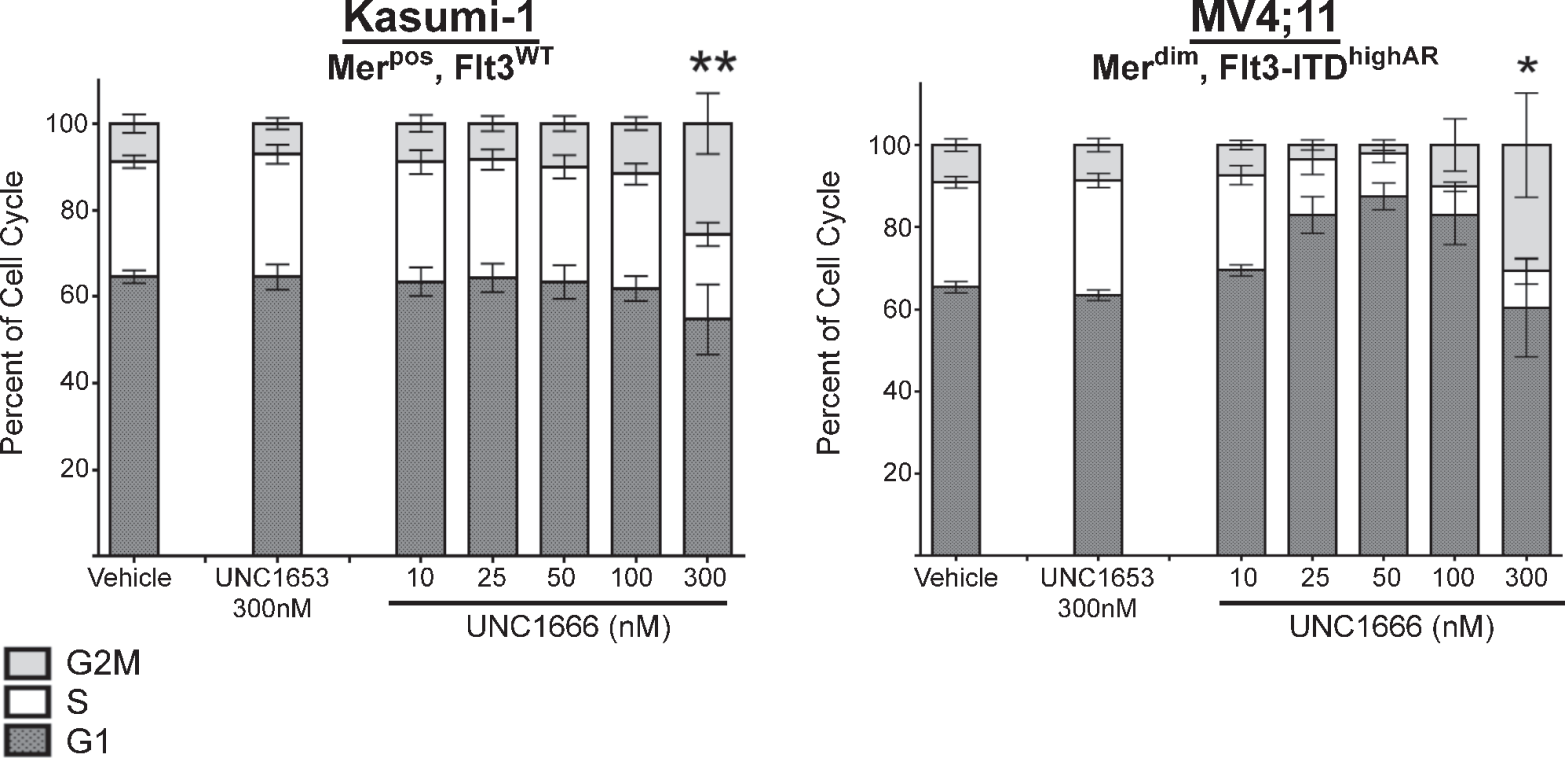
UNC1666 results in G2/M cell cycle arrest at higher concentrations Mer positive or Flt3-ITD AML cell lines were treated with UNC1666, vehicle (DMSO), or inactive control TKI UNC1653 for 72 hours, fixed with 100% ethanol and then analyzed by flow cytometry after staining with propidium iodide to identify stage of cell cycle. Graphic representation of cell cycle progress in Kasumi-1 and MV4;11 cells using ModFit analysis are shown. The percentages of cells in G2/M (light gray), S (white), and G1 (dark gray) phases are shown. Mean values and standard errors were derived from at least 3 independent experiments. *p < 0.05, **p < 0.01.

To analyze the functional effects mediated by UNC1666, AML cell lines were treated with UNC1666, vehicle or negative control UNC1653 for 72 hours and cells were analyzed by flow cytometry after staining with YO-PRO-1 iodide and propidium iodide dyes, compounds that are taken up by early apoptotic or late apoptotic/dead cells, respectively (Figure 3A). Treatment of Mer-positive cell lines with UNC1666 resulted in a dose-dependent induction of apoptosis relative to vehicle-treated cells (Figure 3B & Supplemental Figure 3A). Mer-expressing cell lines exhibited a significant induction of apoptosis in 50–67% of cells at 100 nM and an even more dramatic induction of apoptosis in 68–76% of cells at 300 nM. For example, in the Kasumi-1 cell line 30 ± 7% of cells were apoptotic or dead after 72 hour treatment with vehicle compared to 76 ± 8% after treatment with 300 nM UNC1666 (*p* < 0.001). Importantly, apoptosis was induced at the same concentrations of UNC1666 required for inhibition of Mer (Figures 2A and 3B). In Flt3-ITD cell lines, treatment with UNC1666 resulted in an even more dramatic induction of apoptosis (Figure 3B & Supplemental Figure 3A). At 50 nM, 54–67% of Flt3-ITD cells were apoptotic or dead, increasing up to 90–98% at 300 nM. More specifically, the MV4;11 cell line demonstrated 13 ± 3% apoptosis after treatment with vehicle versus 90 ± 1% after treatment with 300 nM UNC1666, and the MOLM-13 cell line demonstrated respective means of 8 ± 1% and 98 ± 2% (both *p* < 0.001). Again, induction of apoptosis correlated with inhibition of Flt3 (Figures 2B and 3B). Treatment with negative control UNC1653 did not induce apoptosis in Mer-expressing or Flt3-ITD cell lines (Figure 3B). Induction of apoptosis was confirmed by immunoblot demonstrating increased cleavage of PARP and Caspase-3 after treatment with UNC1666 (Figure 3C). Cell cycle analysis indicated a trend towards accumulation of cells in G2M phase with 4N DNA content and this was significant at the 300 nM dose of UNC1666 (Figure 4 and Supplemental Figure 3B).

### UNC1666 affects growth & proliferation of AML cells even after treatment

**Figure 5:**
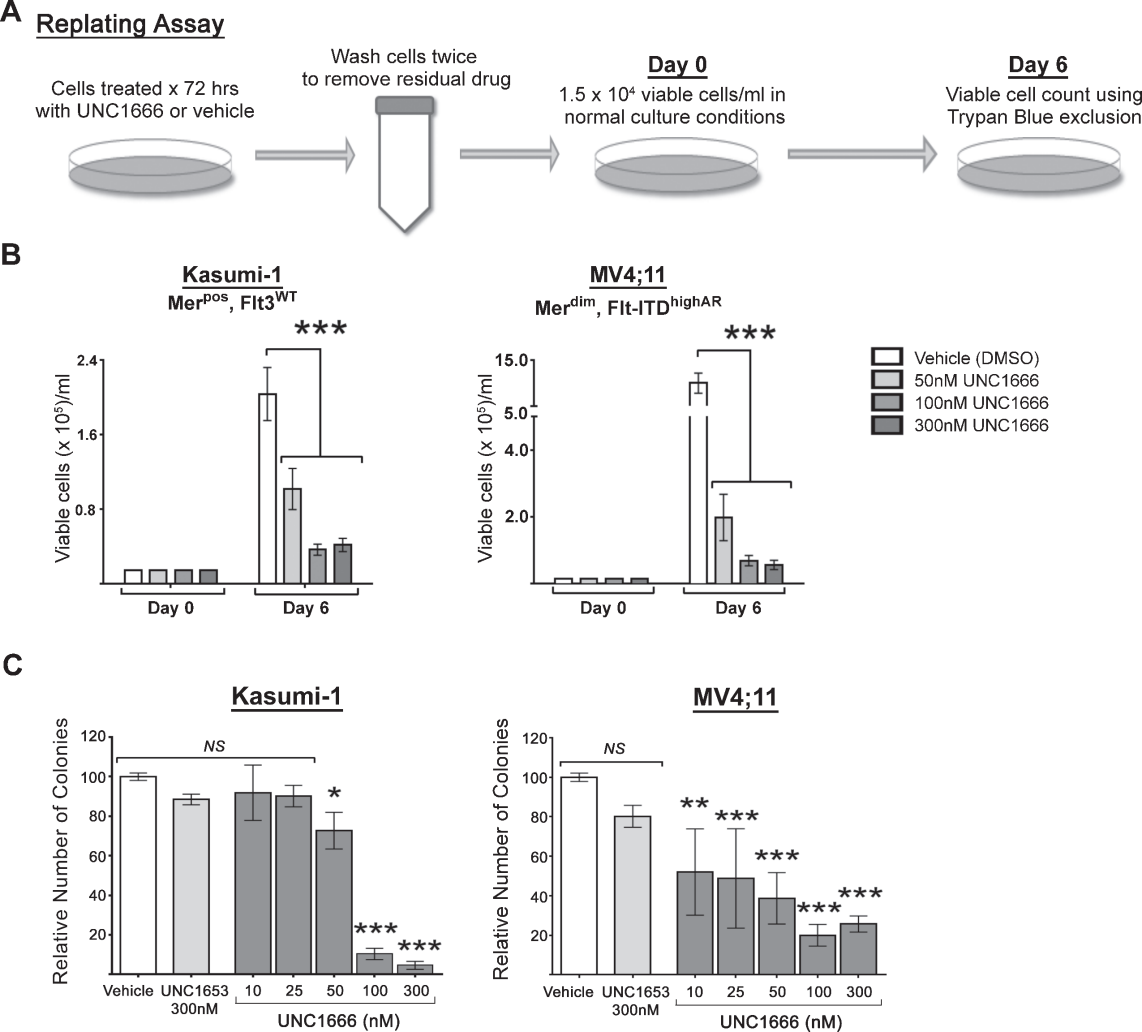
UNC1666 diminishes myeloblast rebound growth and colony formation in AML cell lines expressing Mer or Flt3-ITD **(A)** Diagram of the replating assay. Cells were treated with UNC1666 or vehicle for 72 hours, then washed to remove any residual compound and equal numbers of viable cells (1.5 × 10^4^/ml) were replated in growth medium on Day 0. On Day 6 after replating, the number of viable cells was determined. **(B)** Graphic representation of the results of the replating assay in Mer or Flt3-ITD expressing cell lines demonstrating decreased rebound growth after treatment with UNC1666. Mean values and standard errors were derived from at least 3 independent experiments. **(C)** Colony-formation assays were performed using Mer or Flt3-ITD expressing AML cell lines. Cells were grown in soft agar with the indicated treatments. Graphic representation of reduced colony number after treatment with UNC1666, compared to vehicle or negative control TKI UNC1653. Mean values and standard errors were derived from at least 3 independent experiments. *p < 0.05, **p < 0.01, ***p < 0.001, *NS* = not significant.

Malignant cells have previously demonstrated rebound growth after removal of TKIs in some clinical trials [26]. To evaluate the sustained effect of UNC1666 on cell growth and survival after removal of treatment, AML cell lines were cultured with UNC1666 or vehicle for 72 hours, then cells were washed and replated at 1.5 × 10^4^ viable cells/ml in normal culture conditions (Day 0). Cells were counted after six days of culture (Day 6) to assess rebound growth (Figure 5A). Although 30–75% of cells remain viable after treatment with 50 nM UNC1666 compared to vehicle (Figure 3B and Supplemental Figure 3A), these cells demonstrate a significant decrease in their ability to proliferate even after UNC1666 removal (Figure 5B and Supplemental Figure 3C). Samples treated with higher concentrations of UNC1666 had even more striking cell proliferation defects, leading to only a minimal number of viable cells at Day 6 in all four cell lines. The Kasumi-1 cell line treated with vehicle demonstrated an average 14-fold increase in viable cell number (from 1.5 × 10^4^ to 2.0 × 10^5^ viable cells/ml) over six days, whereas those treated with 100 nM UNC1666 only increased 2.5-fold (to 0.3 × 10^5^ cells/ml) (*p* < 0.001). The MV4;11 cell line treated with vehicle demonstrated an average 67-fold increase (from 1.5 × 10^4^ to 10 × 10^5^ viable cells/ml) whereas cells treated with 50 nM UNC1666 only increased 13-fold (to 2.0 × 10^5^ viable cells/ml) (*p* < 0.001).

### UNC1666 decreases colony formation in AML cell lines

To assess the effect of UNC1666 in a longer-term assay which might more closely approximate the three-dimensional environment encountered *in vivo*, AML cell lines were plated in equal number in soft agar and treated with UNC1666, negative control UNC1653, or vehicle in the overlaying medium. Vehicle treatment was assessed in duplicate and data from samples treated with UNC1666 or UNC1653 were normalized to the mean vehicle colony number for each experiment. Treatment with UNC1666 significantly decreased colony formation compared to cells treated with medium containing vehicle or UNC1653 (Figure 5C & Supplemental Figure 3D). The Kasumi-1 and NOMO-1 cell lines demonstrated a 90 ± 6% and 71 ± 4% reduction in colony formation, respectively, in response to 100 nM UNC1666, while the MV4;11 and MOLM-13 cells line demonstrated a 61 ± 22% and 93 ± 6% reduction in colonies, respectively, in response to treatment with 50 nM UNC1666 (*p* < 0.001).

### UNC1666 inhibits downstream signaling in primary AML cells

**Figure 6:**
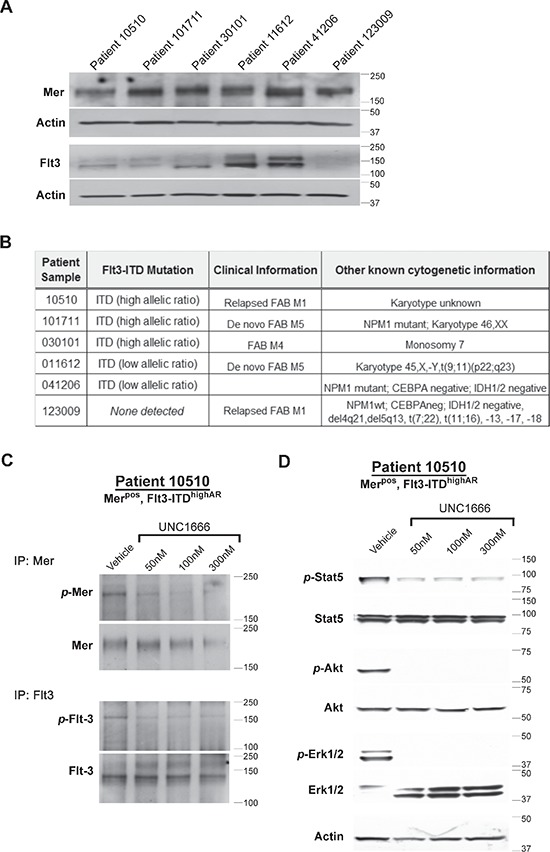
UNC1666 inhibits Mer and Flt3 dependent signaling in AML patient samples **(A)** Immunoblot analysis of Mer and Flt3 expression in lysates prepared from AML patient samples. **(B)** Flt3 mutation status of patient samples determined by molecular profiling. **(C, D)** Dose-dependent inhibition of Mer and Flt3 phosphorylation in response to treatment with UNC1666. AML blasts from patient sample #10510 (Mer positive, Flt3-ITD high allelic ratio) were treated with UNC1666 or vehicle for two hours. **(C)** Mer and Flt3 were immunoprecipitated from cell lysates and phosphorylated Mer (*p*-Mer), total Mer (~180 kDa), phosphorylated Flt3 (*p*-Flt3) and total Flt3 (130/160 kDa) were detected by immunoblot. **(D)** Phosphorylation of downstream signaling molecules was assessed by immunoblot after treatment with UNC1666 or vehicle.

Mer and wild-type Flt3 expression levels were assessed in blasts obtained from six patients diagnosed with acute myeloid leukemia using immunoblot analysis (Figure 6A), and *FLT3-ITD* alleles were detected using standard clinical molecular techniques (Figure 6B). Additional clinical information relevant to each patient samples is described in Figure 6B. In our ongoing studies, we have not identified any cell line other than MV4;11 (which expresses very little Mer) that jointly expresses Mer and a Flt3-ITD mutation (data not shown), however five out of six of our randomly obtained patient samples did co-express these molecules. All samples expressed Mer (consistent with our previously published results), but demonstrated a range of Mer expression levels (Figure 6A). Since sensitivity of Mer expressing malignant cell lines does not appear to directly correlate with the degree of Mer expression (data not shown) and Mer dependence may instead be related to autocrine ligand expression or some other property of the cell, we opted to score all patient samples that express Mer as Mer positive (Mer^pos^). All samples except #123009 expressed Flt3-ITD mutations, though #11612 and #41206 were noted to have a low allelic ratio. Since there is a correlation between high allelic ratio of the Flt3-ITD (> 0.4) and poor prognosis in patients [16], we accordingly denoted when a patient sample was of high (“Flt3-ITD^highAR^”) or low (“Flt3-ITD^lowAR^”) Flt3 allelic ratio. None of the patient samples demonstrate detectable levels of Trk proteins (Supplemental Figure 4A). Sample #10510, which was Mer^pos^ and Flt3-ITD^highAR^, was analyzed to determine intracellular signaling alterations after treatment with UNC1666. Mer and Flt3 were immunoprecipitated from whole cell lysates and immunoblot analysis confirmed inhibition of both kinases in response to UNC1666 (Figure 6C). Phosphorylation of both Mer and Flt3 was inhibited by greater that 50% after treatment with UNC1666 at concentrations as low as 50 nM, though it is notable that UNC1666 appears to decrease Flt3 phosphorylation at a lower dose compared to its effect on Mer, similar to what was observed in cell lines. Immunoblot analysis of Erk1/2, Akt and Stat5 phospho-proteins demonstrated marked decreases in response to UNC1666 treatment (Figure 6D), which correlated the decrease in Mer and Flt3 phosphorylation status.

### UNC1666 induces apoptosis in primary AML patient samples

**Figure 7:**
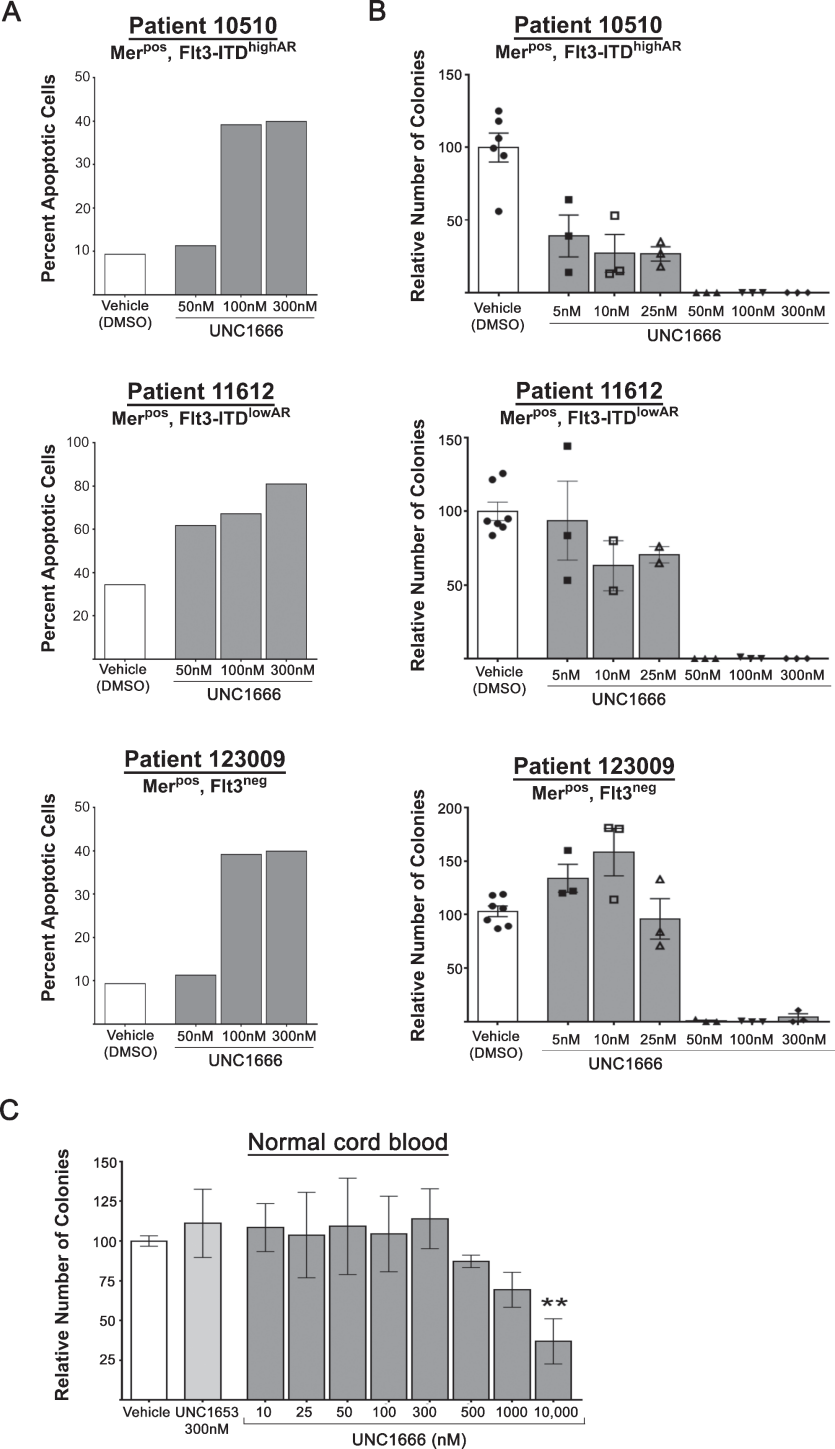
UNC1666 induces apoptosis and decreases colony formation in AML patient samples **(A)** Graphic representation of apoptosis and cell death in AML patient samples after treatment with UNC1666 or vehicle for 72 hours. Apoptotic and dead cells were determined by flow cytometry after staining with YO-PRO-1 iodide and propidium iodide. Values derived from each sample are shown. **(B)** Colony-forming assays were performed in methylcellulose with the indicated treatments. Graphic representation of reduced colony number after treatment with UNC1666, compared to vehicle. Mean values and standard errors derived from triplicate samples are shown. **(C)** Graphic representation of the effect of UNC1666 on normal cord blood colony forming potential. Mean values and standard errors were derived from 3 independent experiments. **p < 0.01, *NS* = not significant.

To replicate the conditions utilized with AML cell lines, patient samples were treated with UNC1666 or vehicle and apoptosis was determined using flow cytometry as described above. Patient samples were co-cultured with HS27 stromal cells, which provide essential factors to support myeloid cells and allowed us to assess response to UNC1666. Under the stromal cell enhanced growth conditions, treatment with UNC1666 was sufficient to induce apoptosis in all patient samples, thereby demonstrating efficacy on AML blasts with a wide range of Mer expression levels or Flt3-ITD status (Figure 7A & Supplemental Figure 4B). Induction of apoptosis in response to UNC1666 was independent of position in the cell cycle, though at high doses may correlate with accumulation in G2/M phase as previously noted in AML cell lines (Supplemental Figure 4C).

### UNC1666 decreases colony formation in methylcellulose in primary AML patient samples

To determine the effects of UNC1666 on colony-forming potential, AML patient samples were cultured in methylcellulose with the compound or vehicle for 10 days and colonies were counted in a blinded manner. Patient samples grew variably under these conditions, so experimental data are reported as colony number relative to vehicle-treated cultures to facilitate comparison between samples. UNC1666 treatment dramatically decreased colony numbers in all patient samples and mediated near complete abrogation of colony formation at concentrations as low as 50 nM in all but one sample (Figure 7B & Supplemental Figure 4D).

### UNC1666 provides a broad therapeutic dosing window in normal hematopoietic precursors

A similar evaluation was performed to determine the effects of treatment with UNC1666 or vehicle control on hematopoietic precursors in methylcellulose cultures of normal umbilical cord blood samples. In these studies there was no significant reduction in colony number at doses up to 500 nM UNC1666 (*n* = 3, Figure 7C). Upon treatment with 1 μM UNC1666, there is a trend towards decreased colony formation, and this becomes statistically significant at a dose of 10 μM.

## DISCUSSION

The unacceptable cure rate and toxic consequences of intensive chemotherapy support the development of novel targeted therapies to improve long-term clinical outcomes in AML. Our previous work demonstrated the upregulation of the Mer tyrosine kinase in nearly all adult and pediatric patient samples collected at diagnosis or relapse. In contrast, normal bone marrow progenitors express very little Mer [8, 9]. We previously found that inhibition of Mer using RNA interference induced apoptosis and decreased proliferation *in vitro* and prolonged survival *in vivo* in xenograft models. These results indicated a potential therapeutic role for Mer kinase inhibition. In an effort to discover Mer-selective inhibitors, we generated UNC1666, which also inhibits Flt3 at nearly equal potency in enzymatic assays. The sequence identity between the binding pockets of Mer and Flt3 (defined as all residues whose side chains are within 4.5 Å distance from UNC1666) is > 90% (one differing residue). In contrast, the sequence identity for the full Mer kinase domain compared to the full kinase domain of Flt3 is ~30%. UNC1666 exhibits selectivity for Mer and Flt3 over other kinases tested, with greater than 50-fold selectivity over closely related and structurally similar kinases Tyro-3 and Axl, co-members with Mer in the TAM family.

We have found that Mer and Flt3-ITD can be co-expressed on AML patient samples, though a larger cohort of patient samples is needed to determine the frequency of dual Mer expression and Flt3-ITD mutation. Currently, little is known about the physical association of Mer and Flt3 receptors on leukemic blasts. Though studies to assess the physical association of Mer and Flt3 have not been reported, co-localization of Flt-3 and Axl, a member of the TAM kinase family along with Mer has been demonstrated [27]. Based on the structural homology of Mer and Axl, it is possible that Mer and Flt-3 might also co-localize and perhaps directly associate.

Given that Flt3-ITD is a known oncogenic target in AML and Mer is also known to play essential oncogenic roles in AML cells, combined inhibition of Mer and Flt3 is likely to be an effective strategy with broad applicability. Thus, we tested the efficacy of our dual Mer/Flt3 TKI in AML cell lines and patient samples. The MCE enzymatic assay data indicate that UNC1666 inhibits Mer and Flt3 with nearly equal potency. However, in cell-based assays, UNC1666 was more effective at reducing Flt3 phosphorylation compared to Mer phosphorylation. Inhibition of Mer and Flt3 phosphorylation in cell based assays correlated with functional effects mediated by UNC1666 in cell lines expressing Mer or Flt3-ITD.

Previously, we demonstrated signaling downstream of Mer through the pro-survival and anti-apoptotic Erk1/2, Akt and Stat6 pathways in AML. Other groups have shown similar downstream effectors of Flt3-ITD including Erk1/2, Akt and Stat5. Though there are have been additional effectors known downstream of both Mer and Flt3 in various cell types, we chose to analyze these molecules which function to promote cancer cell growth and prevent apoptosis and have well-characterized roles in leukemogenesis. In addition, other groups have noted heterogeneity in the phosphorylation status of all three of these molecules (in both Mer and/or Flt3-ITD dependent cell lines) depending on the cell line, tumor type, assay, and/or *in vitro* therapeutic intervention. Given the heterogeneity of downstream effector phosphorylation status in a given AML cell line or patient sample, blockade of both Mer and Flt3 signaling may affect independent downstream pathways and/or may function redundantly to more effectively inhibit shared pathways. Targeting these mutual pathways had a profound functional effect in AML patient samples that co-expressed Mer and Flt3, including decreased ability to evade apoptosis and decreased colony formation. Each of these effects occurred at concentrations between 5–100 nM, and correlated well with inhibition of Mer and Flt3 phosphorylation in cell-based assays. The observed correlations between inhibition of Mer or Flt3 and functional anti-leukemia effects mediated by UNC1666 in AML cells suggest that these effects are a result of on-target inhibition of Mer and Flt3, rather than non-specific inhibition of other kinases.

The studies presented here extend the action of UNC1666 to primary patient AML blasts. Interestingly, the anti-proliferative effects in patient samples were even more dramatic than in cell lines with near complete induction of apoptosis and growth inhibition at 50 nM. The patient sample conditions (co-culture with stromal cells) suggest that Mer inhibition is likely to be effective even in the bone marrow microenvironment. The bone marrow niche provides a potential rich source of paracrine Gas6 [28, 29], a Mer ligand, and the ability of Mer inhibitors to abrogate this niche survival pathway may be crucial in improving clinical outcomes in patients. Interestingly, in cell lines treatment with UNC1666 did not have a significant effect on the cell cycle unless high concentrations (300 nM) were used. This effect was also observed in a subset of patient samples, although less consistently. This suggests that blast survival is more profoundly affected than proliferation, and that inhibition of Mer or Flt3 decreases survival at any stage of the cell cycle. Additionally, although patient-derived myeloblasts are sensitive to UNC1666 in methylcellulose cultures at concentrations as low as 5–50 nM, it is important to note that UNC1666 did not affect the colony forming potential of hematopoietic progenitors in normal cord blood cultures, even at doses as high as 1 μM. These data suggest the possibility of minimal bone marrow progenitor toxicity in response to treatment with a dual Mer/Flt3 inhibitor such as UNC1666, even at doses considerably higher than are necessary to decrease receptor phosphorylation and inhibit oncogenic phenotypes and indicate a wide therapeutic window with minimal hematopoietic side effects at doses up to 1 μM. Hematopoietic progenitors have previously been described as Mer negative [8, 9]. Therefore, a Mer targeted inhibitor acting in the bone marrow microenvironment is likely to target only Mer-expressing leukemia cells.

Initially, UNC1666 was designed to have greater solubility to improve *in vivo* bioavailability and pharmacokinetics as compared to earlier Mer inhibitors [23, 30]. Unfortunately, following intravenous administration in a murine model, UNC1666 demonstrated a terminal elimination half-life of 0.23 hour, and following oral administration of UNC1666, the absolute oral bioavailability was low (8%). Due to these observations, we have continued optimization to seek compounds that inhibit Mer and Flt3 with extended plasma half-life and improved bioavailability. However, the studies presented here provide an important proof-of-principal demonstrating that inhibition of Mer or Flt3 has a profound effect on the survival and continued proliferation of myeloblasts and suggest that inhibitors targeting both of these kinases may be particularly effective and/or have broad-spectrum clinical application in patients with AML. Given that our *in vitro* work with primary patient samples demonstrated a dramatic effect at concentrations well within the range of what is clinically achievable using orally bioavailable TKIs, we anticipate that improved analogues will have similar results both *in vitro* and *in vivo*.

Because UNC1666 has poor *in vivo* bioavailability and a short half-life (data not shown), it was not possible to assess the toxicity profile associated with dual Mer/Flt3 inhibition in mice. As previously described, Flt3 inhibitors such as AC-220 are known have side effects including myelosuppression, though this is variable and is not present with some newer generation Flt3 inhibitors [31] due to improved specificity and decreased off-target effects. We would anticipate that UNC1666 would have a side effect profile similar to these newer generation inhibitors given that they have high specificity for Flt3. Additionally, Mer is not expressed in normal marrow progenitors [8, 9]. Previous analysis by our group on bone marrow progenitors in Mer knockout mice show there is a slight decrease in granulocyte-macrophage populations compared to wild-type mice, which is of questionable clinical significance [9]. Other progenitor or stem cell populations were not affected in these studies. In pedigrees describing humans with complete Mer protein loss-of-function, the identified cases lived to adulthood, though they develop clinical conditions such as retinitis pigmentosa, which is thought to be mainly a result of the long-term defects in Mer-dependent clearance of apoptotic cells in the retina. In rat models of retinitis pigmentosa with Mer gene deletion, when the Mer gene was exogenously expressed in the retina, the disease phenotype was corrected [32], indicating that the condition was not permanent. These observations suggest that short-term use of a Mer inhibitor for cancer therapy is unlikely to have an effect as severe as the phenotype observed in patients with Mer loss-of-function mutations and that any observed effects will be reversible. These observations suggest that short-term use of a Mer inhibitor for cancer therapy is unlikely to have an effect as severe as the phenotype observed in patients with Mer loss-of-function mutations and that any observed effects will be reversible. While our studies using cord blood progenitors and the phenotypes associated with loss of Mer function suggest that Mer inhibition will be well-tolerated, there are no Mer selective inhibitors that have been tested in vertebrates and thus toxicity profiles have not been established.

Despite our finding that the majority of Mer-expressing cell lines have at least some degree of response to treatment with UNC1666 in apoptosis and colony formation assays, the level of Mer protein does not correlate with sensitivity to UNC1666. This observation has recently led to initiation of studies to investigate the characteristics of AML cells that might predict sensitivity to Mer inhibition. Based on previous studies indicating a prognostic role for Gas6 in AML, we hypothesize that paracrine or autocrine expression of Gas6 (or other Mer ligands) by the leukemia cell itself or by the supportive marrow stroma [29, 33], could serve as a clinical biomarker, however further investigation is needed.

In summary, Mer and Flt3 inhibition was effective at profoundly diminishing survival of AML blasts in both cell lines and primary patient samples. UNC1666 decreased Mer and Flt3 phosphorylation/activation and abrogated downstream signaling through the Stat, Akt and Erk pathways. The dose required for these signaling effects correlated well with induction of apoptosis and decreased colony formation. Prolonged effects were even noted on seemingly viable cell populations in replating assays, a promising indication for future *in vivo* experimentation with more bioavailable compounds that share UNC1666′s improved potency. These data also suggest a potential role for dual Mer/Flt3 inhibition in the treatment of human AML, where there is a true need for targeted agents to treat patients more effectively.

Interestingly, several additional cancers are known to aberrantly express Mer, including acute lymphoblastic leukemia and melanoma, and inhibition of Mer in these cancers has demonstrated significant effects on malignant cell survival and apoptosis [9, 34]. These malignancies are also likely to benefit from the development of newer generation Mer inhibitors with improved *in vivo* bioavailability for use in the treatment of human disease, though it is not clear if these cancers would equally benefit from the dual Mer/Flt3 inhibition. The data presented here are likely to lead to a multitude of additional studies of our Mer/Flt3 inhibitors exemplified here by UNC1666, including evaluation of potential synergistic combination therapy, assessment in murine xenograft mice using patient derived xenografts, and ultimately to phase I clinical trials. The hypothesis that Mer is providing the neoplastic cell with a survival advantage and the observation that Mer inhibition sensitizes cancer cells to chemotherapy [9] suggest that combination with cytotoxic therapy may be particularly efficacious, potentially allowing for chemotherapy dose-reduction. In AML, the dual Mer/Flt3 activity of this series of compounds should provide additional efficacy relative to inhibition of either kinase alone. Ultimately, the use of similar inhibitors is anticipated to improve the treatment of AML, with potential to decrease relapse rates and as a treatment option for patients who are not able to tolerate the toxicity of current standard chemotherapeutics.

## METHODS

### Compound development and kinase assays

UNC1666 and UNC1653 (a non-active analog) were prepared as previously described [24, 30]. Kinase inhibition profiling was performed by Carna Biosciences to assess for off-target inhibition mediated by UNC1666. Inhibition constants of Mer, Flt3, Tyro3 and Axl kinase activity by UNC1666 was determined at the Km for ATP using a microfluidic capillary electrophoresis (MCE) assay [35–37] in which phosphorylated and unphosphorylated substrate peptides were separated and analyzed using a LabChip EZ Reader [23, 30]. See Supplemental Methods for additional information. For *in vitro* studies, compounds were dissolved in dimethyl sulfoxide (DMSO; Sigma). DMSO equivalent to the 300 nM UNC1666 treatment was used as a vehicle control.

### Patient samples and cell culture

Cell lines MV4;11, NOMO-1, KG-1a, NB-4, and HEL were obtained from the German Collection of Microorganisms and Cell Culture (DSMZ); Kasumi-1 and U937 were obtained from the American Type Culture Collection (ATCC); MOLM-13 was a gift from Robert Arceci (Johns Hopkins); HS-27 was a gift from Kathrin Bernt (University of Colorado). AML cell lines were maintained in RPMI medium (HyClone) supplemented with 10% FBS and penicillin/streptomycin (cRPMI). Cell line identities were confirmed using short tandem repeat microsatellite loci analysis. HS-27 stromal cells were maintained in DMEM with 10% FBS and penicillin/streptomycin prior to co-culture. De-identified cord blood samples and primary patient myeloblasts apheresed from peripheral blood were obtained from the University of Colorado after written informed consent in accordance with the Declaration of Helsinki. Experiments conformed to regulatory standards as approved by the Colorado Multiple Institutional Review Board. AML patient samples were maintained as previously described [38], with HS-27 stromal co-culture for apoptosis and cell cycle experiments.

### Immunoblot analysis

Three million cells per condition were cultured in 24-well plates and treated with UNC1666, UNC1653, or vehicle for two hours. Whole cell lysates were prepared and proteins were resolved on Tris-Glycine SDS-PAGE gels (Invitrogen) and transferred onto nitrocellulose membranes. Membranes were blocked in tris-buffered saline with 0.1% Tween-20 containing 5% bovine serum albumin (when probing for Flt3) or 5% milk (when probing for all other molecules). For immunoprecipitation assays, cells were plated and treated as above, then treated with pervanadate for 10 minutes prior to cell lysis to stabilize the phosphorylation status. With the use of pervandate, it was not necessary to stimulate cells with exogenous Mer or Flt3 ligands (other than from fetal bovine serum) to detect phosphorylated Mer and Flt3 proteins. Anti-Mer antibody or anti-Flt3 and rec-Protein G-sepharose beads (Invitrogen) were added and lysates were incubated overnight on a rocking platform. Beads were washed and eluted proteins were resolved as above. Immunoprecipitate membranes were probed with an anti-phospho-Mer or anti-phospho-Flt3 antibody. After visualization, membranes were stripped and reprobed for total protein. The following antibodies were used according to manufacturer recommendations: anti-Mer (ab52968, AbCam); anti-phospho-Mer (Phosphosolutions Inc.) [34, 39]; anti-Flt3 (sc-480), anti-Actin (sc-1616), anti-pan-Trk (sc-11), donkey-anti-goat IgG-HRP (sc-2020) (Santa Cruz Biotechnology); anti-phospho-Flt3 (#3461), anti-phospho-Stat6 (Tyr641, #9364), anti-Stat6, (#9362), anti-phospho-Stat5 (Tyr694, #9359), anti-Stat5 (#9358), anti-phospho-AKT (Ser473, #9271L), anti-AKT (#9272), anti-phospho-p44/42-MAPK (ERK1/2, Thr202/Tyr204, #9106), anti-p44/42-MAPK (#9102), anti-PARP (#9542), anti-Caspase-3 (#9665), anti-Tyro-3 (#5585) (Cell Signaling Technology); anti-Axl (AF154, R&D Systems); goat-anti-mouse IgG-HRP, goat-anti-rabbit IgG-HRP (BioRad). Proteins were visualized by horseradish peroxidase chemiluminescence detection (Perkin-Elmer).

### Apoptosis & replating assays

To assess apoptosis, AML cell lines were cultured at 3 × 10^5^ cells, and 1 × 10^6^ AML patient blasts were co-cultured with 3.5 × 10^5^ HS27 cells per condition in 24 well plates, and treated for 72 hours. Harvested cells were washed, resuspended in PBS containing 1 μM YO-PRO^®^-1 iodide (Invitrogen) and 1.5 μM propidium iodide (PI) (Invitrogen), and incubated on ice for 15–20 minutes. Fluorescence was detected and analyzed using a FC500 flow cytometer with CXP data analysis software (Beckman Coulter). For cell cycle analyses, cells were fixed with 100% ethanol after treatment for 72 hours with UNC1666 or controls, stained with PI overnight, and analyzed on a Gallios flow cytometer. Flow cytometry data were analyzed using Modfit Cell Cycle Analysis software (Verity Software House). In replating assays, cells were similarly plated and treated, then harvested cells were washed twice, and 1.5 × 10^4^ viable cells/ml were cultured in cRPMI for six days. Six days after replating, viable myeloblast count was determined by trypan blue exclusion using a Cedex XS Analyzer (Roche).

### Colony formation assays

Cell lines were plated at a density of 1000 cells/ml in 0.35% agar over 0.5% agar base layer. Agar was overlaid with cRPMI containing UNC1666, UNC1653, or vehicle. Colonies were grown for 14 (NOMO-1, MV4;11, MOLM-13) or 21 days (Kasumi-1) prior to staining with 1 mg/ml nitrotetrazolium blue (Sigma-Aldrich). Treatment-containing medium was renewed twice weekly. Patient samples were plated at a density of 1 × 10^6^ cells/ml in MethoCult H4434 Classic Methylcellulose-Based Medium with Recombinant Cytokines for Human Cells (StemCell Technologies) containing UNC1666 or vehicle in triplicate and colonies were grown for 10 days. Human mononuclear cells were isolated from umbilical cord blood samples using Ficoll-Paque PLUS (GE Healthcare Life Sciences). Cells were grown in serum-free IMDM (HyClone) media containing BIT 9500 Serum Substitute (StemCell Technologies), lipoprotein lipase (Millipore), and 2-mercaptoethanol (Sigma) for one hour, then plated in Methocult H4434 methylcellulose containing UNC1666 or vehicle at 2 × 10^6^ cells/mL in triplicate and colonies were grown for 14 days. Cell line and patient sample colonies were counted using a GelCount colony counter (Oxford Optronix) and cord blood colonies were manually counted in a blinded, non-biased manner.

### Statistical analysis

Statistical analyses were performed using GraphPad Prism software (v6.02), comparing UNC1666 and vehicle-treated samples. Cell cycle and replating assays were analyzed using two-way ANOVA. All other data were analyzed using one-way ANOVA. All statistics were corrected using Bonferroni's multiple comparisons test. Results were considered significant when *p* < 0.05.

## SUPPLEMENTAL METHODS


