# Supplementary material for: Efficacy of a Mer and Flt3 tyrosine kinase small molecule inhibitor, UNC1666, in acute myeloid leukemia
Source: Oncotarget. 2015 Feb 10;6(9):6722–36. doi: 10.18632/oncotarget.3156 (PMC4466645; doi:10.18632/oncotarget.3156)
Supplement: Supplementary file 1 [file oncotarget-06-6722-s001.pdf]

SUPPLEMENTAL METHODS

Microfluidic capillary electrophoresis (MCE) assay

Activity assays were performed in a 384-well polypropylene microplate with a final volume of 50µL in 50mM Hepes, pH 7.4 containing 10mM $MgCl_2$, 1.0 mM DTT, 0.01% Triton X-100, 0.1% bovine serum albumin (BSA) (MCE Buffer), with 1.0 µM fluorescent substrate and ATP at Km for each enzyme (Supplemental Table 1). All reactions were terminated by addition of 20 µL of 70 mM EDTA. Phosphorylated and unphosphorylated substrate peptides (Supplemental Table 1) were separated following a 180 min incubation on a LabChip EZ Reader equipped with a 12-sipper chip in separation buffer supplemented with 1 x CR-8 and analyzed using EZ Reader software.

Morrison ki measurement

Inhibition of Mer, Flt3, Tyro3, Axl and TrkA kinases by UNC1666 was measured in the MCE assay using the Morrison Tight-Binding Method (35). Reactions were performed in a 384-well polypropylene microplate in a final volume of 80 µL in MCE Buffer with 5.0 µM ATP, 2.0 nM Mer, and 1.0 µM peptide substrate. Compounds were tested in 20 dilution (1.5-fold) dose curves spanning a concentration range of 60 nM to 0.027 nM. The compound in 10% DMSO (8.0 µL) was added to the enzyme (40 µL, 4 nM Mer) and allowed to incubate for 10 min followed by addition of 32 µL of substrate mix (containing 2.5 µM peptide substrate and 12.5 µM ATP).

Kinetic reads were taken from 7.0 to 209 min, 12 reads total, on an EZReader, using upstream voltage = −2250V, downstream voltage = −500V and pressure = −1.0 psi (sip time 0.2 sec, buffer 30 sec). The steady-state velocity was analyzed by linear regression of the peptide as percent-conversion/min, and a plot of nM compound (UNC1666) vs. velocity was fit to the Morison equation using GraphPad Prism (v5.01, GraphPad Software), Y = Vo*(1-((((Et+X+(Ki*(1+(S/Km))))-(((Et+X+(Ki*(1+(S/Km))))^2)-4*Et*X)^0.5))/(2*Et))). The total enzyme concentration (Et) was fixed to 0.855 nM as determined from previous titration studies (data not shown) (35). (Y = velocity, X = inhibitor concentration).

Cell cycle analysis of patient samples

To assess cell cycle, 1×10^6 AML patient blasts were co-cultured with 3.5×10^5 HS27 cells in 24 well plates, and treated for 72 hours. After this incubation period, cells were cultured with 10 µM ErdU for two hours, and harvested cells were treated with the Click-iT EdU Alexa Fluor 647 Flow Cytometry Assay Kit (Life Technologies) according to manufacturer instructions. Fluorescence was detected and analyzed using a FC500 flow cytometer with CXP data analysis software (Beckman Coulter).

SUPPLEMENTARY FIGURES AND TABLES

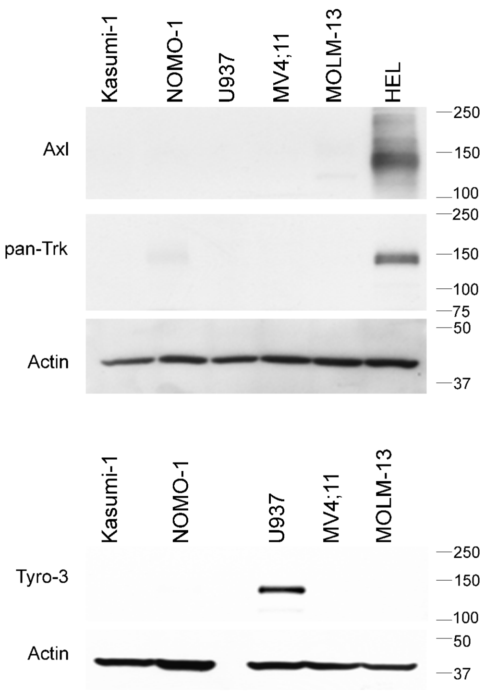

Supplementary Figure 1: Tyro-3, Axl and Trk expression in AML cell lines. (A) Whole cell lysates from AML cell lines were analyzed by immunoblot and demonstrate little to no Axl, Trk or Tyro-3 protein levels in the Kasumi-1, NOMO-1, MV4;11 or MOLM-13 cell lines. HEL (which is known to express Axl and Trk proteins) and U937 (which is known to express Tyro-3) are present as positive controls. Actin is shown as a loading control. Representative blots are shown.

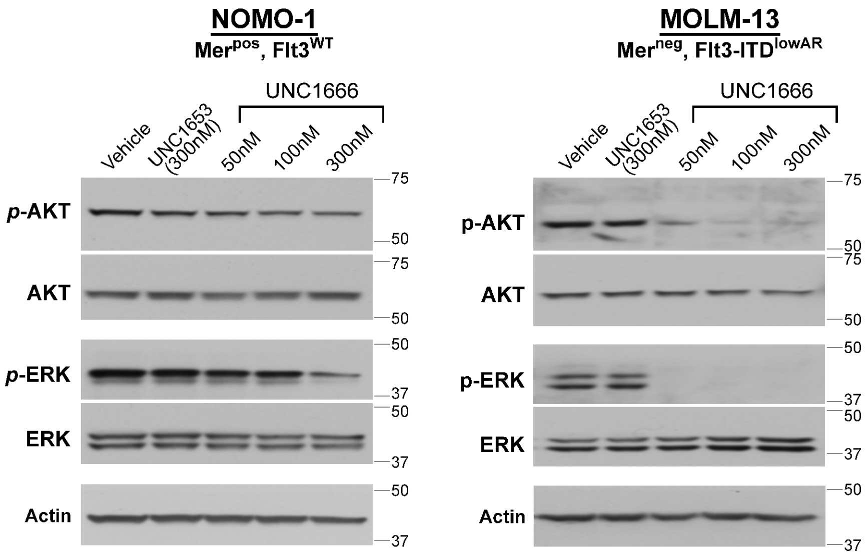

Supplementary Figure 2: UNC1666 reduces Mer and Flt3-mediated anti-apoptotic and pro-survival downstream signaling. (A) Inhibition of downstream signaling after administration of UNC1666 in a Mer-expressing AML cell line (NOMO-1) and a Flt3-ITD mutant cell line (MOLM-13) compared with equivalent concentrations of vehicle (DMSO) or inactive control TKI UNC1653. Actin is shown as a loading control. Representative blots are shown. nM = nanomolar.

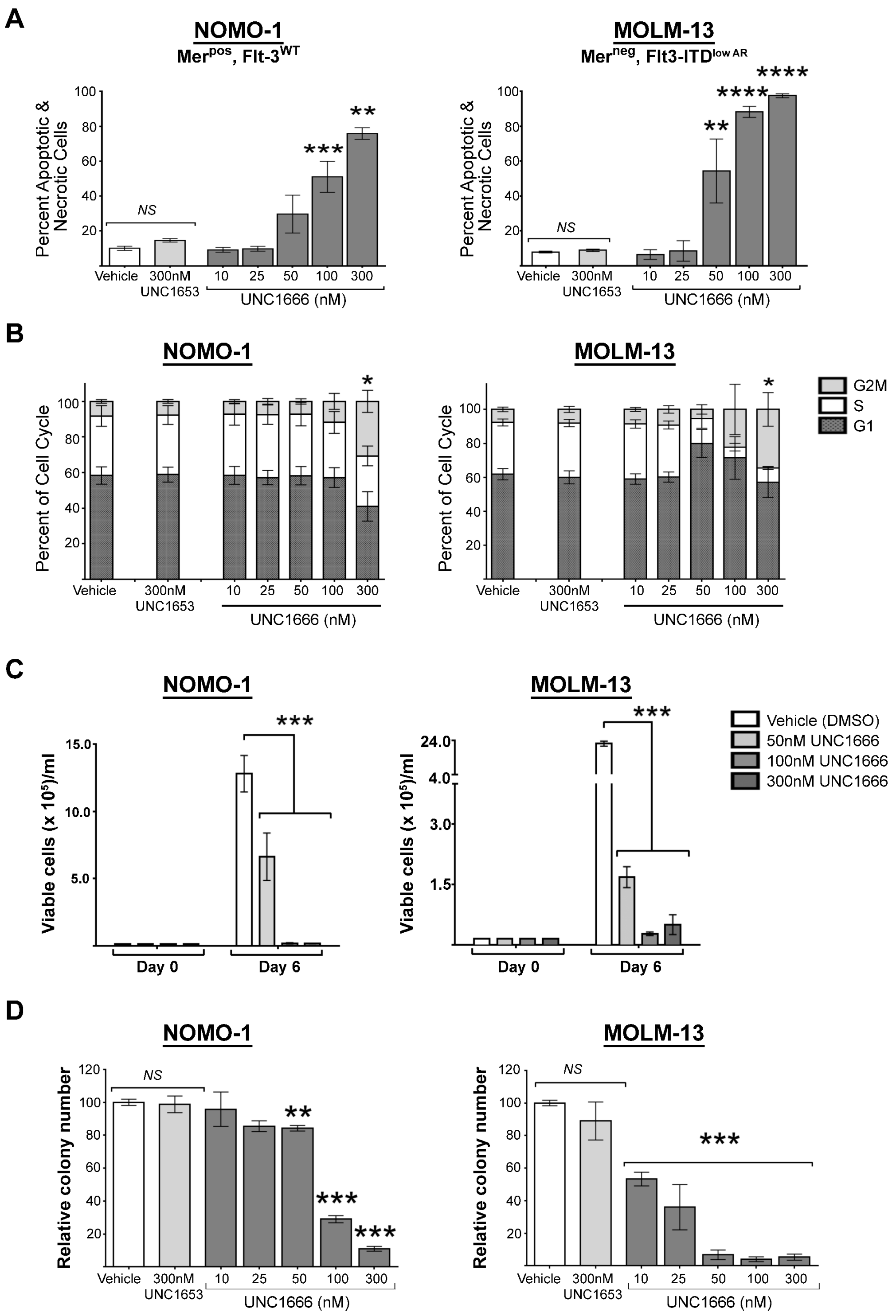

Supplementary Figure 3: UNC1666 induces apoptosis, decreases clonal expansion, and inhibits colony-formation in cultures of myeloblasts expressing Mer or Flt3-ITD. Mer or Flt3-ITD expressing AML cell lines were treated with UNC1666, vehicle (DMSO), or inactive control TKI (UNC1653) for 72 hours and then analyzed by flow cytometry as described in Figures 3A and 4, replated in complete medium to determine recovery from treatment as described in Figure 5A, or cultured in semi-solid medium as described in Figure 5C. **(A)** Graphic representation of flow cytometric analyses of apoptotic/dead cells demonstrating induction of apoptosis. **(B)** Graphic representation of cell cycle progress in NOMO-1 and MOLM-13 cells using ModFit analysis are shown. The percentages of cells in G2/M (light gray), S (white), and G1 (dark gray) phases are shown. **(C)** Graphic representation of the results of the replating assay demonstrating dose-dependent failure to recover from treatment with UNC1666. **(D)** Mer or Flt3-ITD expressing AML cells were grown in soft agar with the indicated treatments. Graphic demonstration of reduced colony number in response to treatment with UNC1666, compared to vehicle or inactive control TKI. Mean values and standard errors were derived from at least 3 independent experiments. *$p < 0.05$, **$p < 0.01$, ***$p < 0.001$, *NS* = not significant.

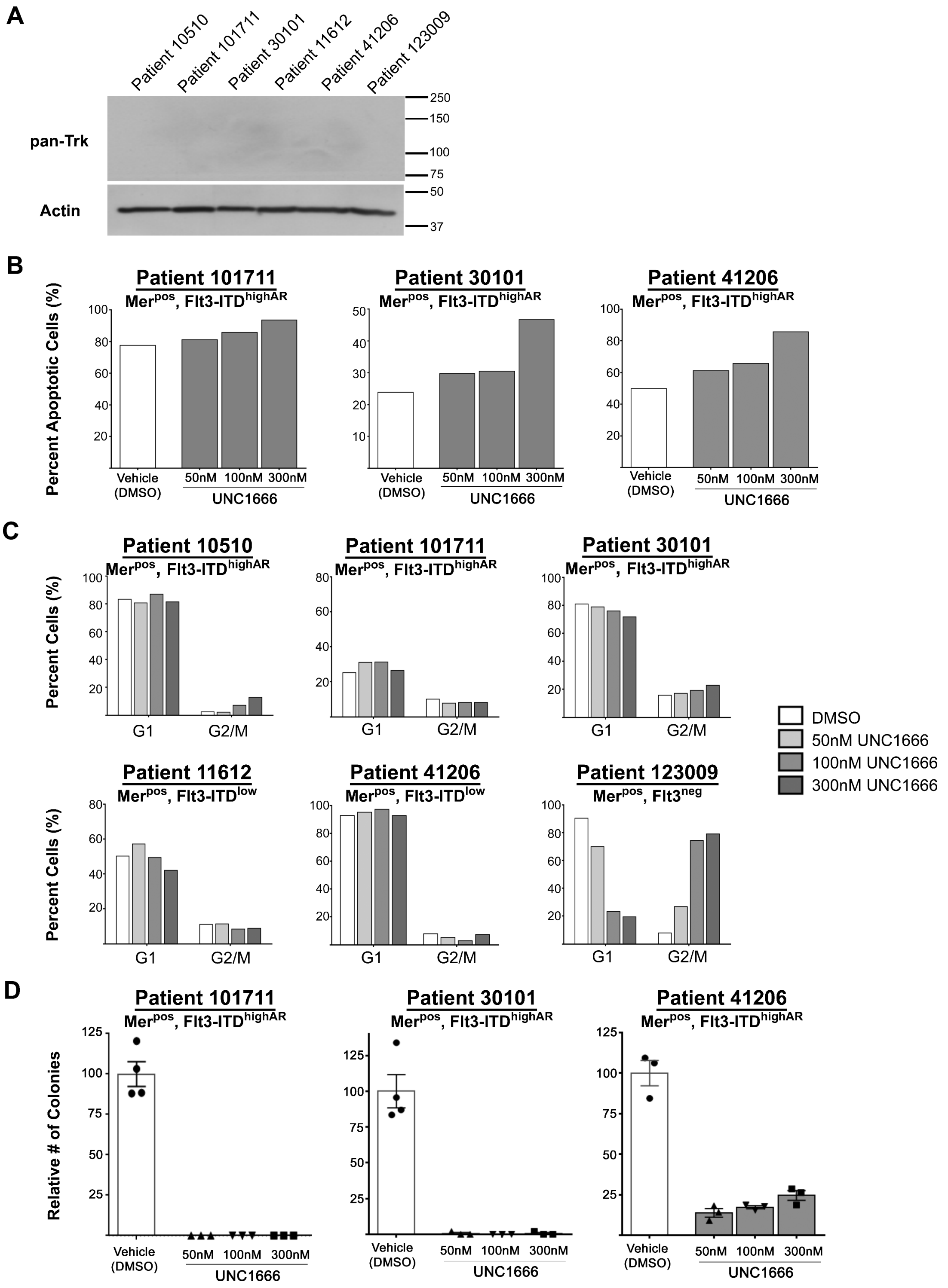

Supplementary Figure 4: UNC1666 induces apoptosis and decreases colony formation in AML patient samples, but does not affect cell cycle control. **(A)** Patient samples were analyzed by immunoblot and demonstrated little or no Trk protein levels. **(B)** Graphic representation of apoptosis and cell death in AML patient samples after treatment with UNC1666 or vehicle for 72 hours. Apoptotic and dead cells were determined by flow cytometry after staining with YO-PRO-1 iodide and propidium iodide. Values derived from each sample are shown. **(C)** Patient samples expressing Mer and/or FLT3-ITD were treated with UNC1666 or vehicle for 72 hours and analyzed by flow cytometry for alterations in cell cycle after staining with propidium iodide. Graphic representation of the percentage of cells in each phase of the cell cycle for each patient sample is shown. **(D)** Colony-forming assays were performed in methylcellulose with the indicated treatments. Graphic representation of reduced colony number after treatment with UNC1666, compared to vehicle. Mean values and standard errors derived from triplicate samples are shown.

Supplementary Table 1: Assay conditions for MCE assays

Kinase	Peptide Substrate	Kinase (nM)	ATP (uM)
Mer	5-FAM-EFPIYDFLPAKKK-$CONH_2$	2.0	20
Axl	5-FAM-KKKKEEIYFFF-$CONH_2$	120	200
Tyro	5-FAM-EFPIYDFLPAKKK-$CONH_2$	10	40
Flt3	5-FAM-KKKKEEIYFFF-$CONH_2$	0.3	275

Supplementary Table 2: Protein kinase profiling panel for inhibitor screening by Carna Biosciences and IC50 values for top targets of UNC1666

Kinase	Percent Inhibition at 46 nM	IC50 value
AXL	87	37 nM
**FLT3**	**101**	**0.69 nM**
**MER**	**103**	**0.55 nM**
TRKA	101	0.57 nM
TRKB	95	
TRKC	98	
TYRO3	73	29 nM
